# Evaluation of Silicon and Proline Application on the Oxidative Machinery in Drought-Stressed Sugar Beet

**DOI:** 10.3390/antiox10030398

**Published:** 2021-03-06

**Authors:** Muneera D. F. AlKahtani, Yaser M. Hafez, Kotb Attia, Emadeldeen Rashwan, Latifa Al Husnain, Hussah I. M. AlGwaiz, Khaled A. A. Abdelaal

**Affiliations:** 1 Biology Department, College of Science, Princess Nourah Bint Abdulrahman University, Riyadh 102275-11675, Saudi Arabia; mdfkahtani@gmail.com (M.D.F.A.); Dr.lathus@gmail.com (L.A.H.); Hussahgwaiz@gmail.com (H.I.M.A.); 2 Excellence Center (EPCRS), Plant Pathology and Biotechnology Lab, Faculty of Agriculture, Kafrelsheikh University, Kafr Elsheikh 33516, Egypt; yasser.abdelgwad@agr.kfs.edu.eg; 3 Center of Excellence in Biotechnology Research, King Saud University, Riyadh 2455-11451, Saudi Arabia; kattia1.c@ksu.edu.sa; 4 Rice Research & Training Center, Rice Biotechnology Lab, Field Crops Research Institute, Sakha, Kafr EL-Sheikh 33717, Egypt; 5 Agronomy Department, Faculty of Agriculture, Tanta University, Tanta 31527, Egypt; emadrashwan@gmail.com

**Keywords:** phenolic compounds, antioxidant enzymes, sugar beet, reactive oxygen species, drought

## Abstract

Drought stress deleteriously affects growth, development and productivity in plants. So, we examined the silicon effect (2 mmol) and proline (10 mmol) individually or the combination (Si + proline) in alleviating the harmful effect of drought on total phenolic compounds, reactive oxygen species (ROS), chlorophyll concentration and antioxidant enzymes as well as yield parameters of drought-stressed sugar beet plants during 2018/2019 and 2019/2020 seasons. Our findings indicated that the root diameter and length (cm), root and shoot fresh weights (g plant^−1^) as well as root and sugar yield significantly decreased in sugar beet plants under drought. Relative water content (RWC), nitrogen (N), phosphorus (P) and potassium (K) contents and chlorophyll (Chl) concentration considerably reduced in stressed sugar beet plants that compared with control in both seasons. Nonetheless, lipid peroxidation (MDA), electrolyte leakage (EL), hydrogen peroxide (H_2_O_2_) and superoxide (O_2_^●−^) considerably elevated as signals of drought. Drought-stressed sugar beet plants showed an increase in proline accumulation, total phenolic compounds and up-regulation of antioxidant enzymes catalase (CAT) and superoxide dismutase (SOD) activity to mitigate drought effects. Si and proline individually or the combination Si + proline considerably increased root and sugar yield, sucrose%, Chl concentration and RWC, MDA and EL were remarkably reduced. The treatments led to adjust proline and total phenolic compounds as well as CAT and SOD activity in stressed sugar beet plants. We concluded that application of Si + proline under drought stress led to improve the resistance of sugar beet by regulating of proline, antioxidant enzymes, phenolic compounds and improving RWC, Chl concentration and Nitrogen, Phosphorus and Potassium (NPK) contents as well as yield parameters.

## 1. Introduction

Sugar beet is an important industrial sugar crop and one of the most significant crops for sugar production, it is adjusted to the Egyptian environment and has an important position in winter crop rotation in all kinds of soils. In Egypt the cultivated area during 2018/2019 season was 255,725.6 hectares producing about 12,247,170 tons [1]. Sugar beet needs a shorter period of productivity and consumes less water than sugarcane [2,3]. Many environmental stress factors affect the plant growth and productivity in various plants; biotic factors [4,5,6,7], salinity [8,9,10,11], heat [12], drought stress [13,14,15]. Drought is one of the most harmful stresses which, threat agricultural production, under drought factor, morphological characteristics such as leaves number, leaf area and stem length were decreased [16,17,18]. Electrolyte leakage (EL)%, lipid peroxidation (MDA) and reactive oxygen species (ROS) were dramatically increased as indicators under drought conditions [19,20]. ROS normally exist in the plant cells with very low concentrations, but under abnormal and stressful conditions, a high accumulation of ROS was observed [21,22,23,24]. Moreover, Chl a, Chl b, relative water content (RWC) and yield components were decreased under drought stress [17,18]. Drought causes a decrease in nutrient uptake from the root system and translocation to the leaves [25], under such a situation, the metabolism is restricted and finally decreased yield production [26]. In sugar beet, drought is the main reason for yield losses because of the accumulation of ions and solutes [27,28]. Enzymatic activity such as antioxidant enzymes catalase (CAT), superoxide dismutase (SOD) and non-enzymatic antioxidants like carotene and ascorbic acid are elevated in stressed plants to scavenge ROS and protect the cells from oxidative stress [29]. Under up-normal circumstances, plants have adaptive mechanisms to acclimate with these harmful conditions during osmotic adjustment, which improve water status and increase organic and nonorganic solutes, consequently, mitigate the drought effects [30,31]. Plants usually have a protective and defense system to alleviate oxidative stress by inducing phenolic and flavonoid compounds under stress [32,33]. Phenolic compound accumulation has been recorded under stress in many plants because of their positive role in alleviating the adverse effects in detoxifying ROS under abiotic and biotic stresses [34]. Siracusa et al. [35] observed an increase in polyphenolic and flavonoid compounds in buckwheat under drought stress. Drought stress boosts vitamin C content, beta-carotene, total polyphenol content (TPC), total antioxidant capacity (TAC) and total flavonoid content (TFC) in the *Amaranthus* plant [36]. Additionally, proline accumulation is extensively reported in drought-stressed plants and under salinity conditions [9,37]. Proline is a very important amino acid and considered as osmoregulators, playing an essential role in osmoregulation to mitigate the injurious impact of stresses such as drought, consequently, improve growth and physiological characters such as Chl concentration, RWC and yield production [16,38]. Exogenously application of proline led to enhance plant growth under stress in calendula and barley plants and alleviate the oxidative damage by reducing the harmful impact of ROS [9,38,39]. It has been stated that under abiotic stress, proline application induces alterations at the structural and ultrastructural levels in the stressed plants, such as improve plant root surface as a strategy to deal with water and nutrient shortage [40]. Interestingly, the positive effect of proline was observed in increased roots number and induced structural alterations in stems and leaves in rice plant under salt stress [41], additionally, the application of proline increased water relations and yield of sugar beet under water drought [42]. It is well established that Si is an essential element in increasing drought stress tolerance and alleviate the mineral nutrient shortage in plants [43]. Si application led to increase root and shoot biomass of tomato [44] and barley plants [45] under drought and in pepper under salinity [8]. Furthermore, Si causes an increase in chlorophyll content, RWC and postponed leaf senescence [46], also, Si can improve the activities of some essential enzymes in rice seedlings under salinity, which scavenge ROS [47] and decrease the oxidative stress of arsenic (As) on wheat seedlings [48]. In this regard, Si may induce secondary metabolism in plants mainly, phenolic compounds in the exposed plants to various stresses [49,50]. Silicon application is an essential approach to reduce the harmful impacts of drought in barley plants [38] and salinity in sweet pepper [8], resulting in improvement in growth characteristics, such as, leaves number, chlorophyll, RWC and enzymes activity in *Salvia* and plants under salinity [51,52]. Interestingly, few studies have investigated the impact of Si and proline on sugar beet growth and sugar yield under drought. Hence, the aim of our study was to evaluate the influence of Si and proline individually or the combination of Si + proline as low-cost and easy-to-implement drought adaptation strategies on root and sugar yield, sucrose %, Chl, RWC, MDA and Enzymes activity as well as total phenolic compounds in sugar beet plants under drought. We expected that the application of Si and proline would decrease the harmful effects of drought on sugar beet due to enhancement of phenolic compounds, RWC, Chl concentration and scavenge ROS.

## 2. Materials and Methods

### 2.1. Expremints Design and Treatments

Tow field experiments were conducted at Gharbia governorate during two winter seasons 2018/2019 and 2019/2020 to evaluate the impact of silicon and proline on morphological, physio-biochemical and yield parameters of sugar beet plants (*Beta vulgaris* L.) cv. Samba under drought stress (50% field capacity). The physio-biochemical studies were done at PPBL Lab and EPECRS Center, Kafrelsheikh University. Seeds of sugar beet were sown on 2nd and 4th October in both seasons, respectively. Each plot contained 5 rows, 60 cm apart, the spacing hill was 60 × 20 cm. There were five treatments, including control (100% field capacity), drought (50% field capacity), drought + Si (2 mmol), drought + proline (10 mmol), drought + Si + proline (2 mmol + 10 mmol). Application of Si and proline (Foliar sprayed) were applied twice, the first was at 35 days from transplanting (DAS) and the second was at 15 days after the first one, the treatments were arranged in a completely randomized design with four replicates. Experimental soil characters were analyzed [53] and the obtained results were, pH 8.1, N 32.6 ppm, P 10.3 ppm, K 288 ppm, electrical conductivity 1.7 dS m^−1^, soil organic matter 1.8%, sand 17.7%, and silt 35.9%. The samples were taken to determine physio-biochemical characters at 120 DAS, however morphological, yield characters, sugar yield and sucrose% were determined at harvest date (195 DAS).

### 2.2. Morphological and Yield Characters

At the harvesting date (195 DAS) ten plants were randomly selected from each plot to determine the morphological and yield characters. Morphological characters such as root length and diameter (cm), root and shoot fresh weights (g plant^−1^), root yield and sugar yield (ton/ha) were determined.

### 2.3. Estimation of Total Soluble Solids (TSS%), Sucrose% and Sugar Yield (t ha^−1^)

Sucrose% and TSS% were estimated as follows: Total soluble solids (TSS%) was recorded in the juice of fresh roots using a hand refractometer, sucrose% was measured polarimetrically on a lead acetate extract of fresh macerated roots [54] and sugar yield (t/ha) was estimated using the following equation: Sugar yield (t/ha) = Root yield (t/ha) × sucrose /100.

### 2.4. Estimation of Chlorophyll A and B Concentrations

Sugar beet fresh leaves discs (10 discs) were taken and placed in a solution containing 95% ethanol and 80% acetone (*v:v* = 1:2). The samples were kept under dark conditions overnight until the green color disappeared from the leaf tissue. The absorbance was recorded at 663, 645 and 470 nm. Chl concentration was assayed according to Lichtenthaler [55].

### 2.5. Estimation of Relative Water Content (RWC%)

Sugar beet fresh leaves discs (10 discs) (1 cm diameter) were taken to determine the fresh weight, and then the discs were soaked in distilled water for 4 h at 25 °C to determine the turgid weight (TW). Dry weight (DW) was measured after keeping them in a hot-air oven for 24 h at 80 °C. Relative water content (RWC) was measured as follow:

RWC % = (FW − DW)/(TW − DW) × 100(1)

where fresh weight (FW); dry weight (DW); turgid weight (TW) [56].

### 2.6. Estimation of Electrolyte Leakage

Twenty discs (1 cm^2^) of sugar beet leaves were taken and electrical conductivity was recorded to determine EL%. Electrolyte leakage % was determined as follows: first conductivity/last conductivity × 100. [57].

### 2.7. Estimation of Proline

Proline concentration was measured in fresh leaves, 100 mg were taken from the fully expanded leaves for analyses, the samples were homogenized in 10 mL of 3% sulfosalicylic acids and filtered using filter paper. Then, 2 mL of the supernatant were made to react with 2 mL of glacial acetic acid and 2 mL of acid ninhydrin in a test tube at 100 °C for 1 h, then the tube was placed on an ice bath. The mixtures were extracted using 4 mL of toluene and vortexing (15–20 s). The chromophore containing toluene was measured at 520 nm using a spectrophotometer with toluene as blank, proline was determined from a standard curve as µmol g^−1^ FW [58].

### 2.8. Estimation of Lipid Peroxidation (MDA)

MDA was determined on fresh leaves by the procedure of Du and Bramlage [59] as malondialdehyde (MDA), the absorbance was recorded spectrophotometrically at 532, and 600 nm.

### 2.9. Estimation of Antioxidant Enzyme Activity

Frozen sugar beet leaves were used for protein extraction, 0.5 g frozen leaves were ground in liquid nitrogen. Protein extraction was done using 3 mL of buffer containing 50 mM K-phosphate buffer (pH 7.0), 2 mM EDTA, 20 mM ascorbate, and 0.1% (*v/v*) Triton X-100 for CAT (EC 1.11.1.6) or 100 mM K-phosphate buffer (pH 7.8), 0.1 mM EDTA, 14 mM 2-mercaptoethanol, and 0.1% (*v/v*) Triton X-100 for SOD (EC 1.15.1.1) activity. The mixture was centrifuged at 15,000× *g* (4 °C) for 15 min [60]. The activity of CAT was estimated at 240 nm using a spectrophotometer depend on the rate of H_2_O_2_ consumption as mmol min^−1^ mg protein^−1^ [61]. The activity of SOD was estimated by the enzyme capability to prevent the photochemical reduction of nitroblue tetrazolium (NBT) on blue formazan and recorded at 560 nm as mmol min^−1^ mg protein^−1^ [62].

### 2.10. Total Phenolic Compounds Determination

The total content of phenolic compounds was determined in sugar beet leaves by the Folin–Ciocalteu reagent according to Singleton and Rossi [63]. The extract solution (0.1 mL) containing 1000 μg of the extract was mixed with 46 mL distilled water in a volumetric flask and 1 mL Folin–Ciocalteu reagent was added, and the flask was shaken. The mixture was allowed to react for 3 min and 3 mL aqueous solution of 2% Na_2_CO_3_ was added. At the end of incubation at room temperature for 2 h, the absorbance was determined by spectrophotometer at 750 nm, the total content of phenolic compounds was recorded as μg gallic acid equivalent in dry weight material (µg mL^−1^ gallic acid equivalent).

### 2.11. Determination of Nitrogen, Phosphorus and Potassium (NPK)

Sugar beet fresh leaves samples were taken and washed with dilute HCl to remove any adhered particles, then washed with deionized water five times to remove HCl. The samples were left to air-dry on room temperature, then, the samples put into a hot-air oven for 48 h on 70 °C. Then the samples were powdered and placed in plastic bags for analysis. For N, P and K measurement, the samples were digested with HNO_3_:HClO_4_ solution (2:1). Nitrogen content% was determined according to A.O.A.C. [64], whereas Phosphorus content% was measured calorimetrically according to Jackson [65]. Potassium content% was determined using Atomic Absorption according to Page et al. [53].

### 2.12. Esimation of Reactive Oxygen Species (ROS)

Hydrogen peroxide (H_2_O_2_) and Superoxide (O_2_^●−^) are the most common free radicles of ROS, (O_2_^●−^) and (H_2_O_2_) were estimated in sugar beet leaves. In the presence of ice fresh leaf tissues (0.5 g) were blended with 3 mL of K-phosphate (50 mM) buffer with 7 pH at 4 °C. Centrifugation of the amalgam was done for 15 min at 12,000× *g*. From the upper layer of the mixture, 3 mL was taken and blended with H_2_SO4 (20% *v/v*) and TiCl4 (1%), then centrifuged for 15 min at 11,500× *g*. The absorbance was recorded using a spectrophotometer at 410 nm to quantify H_2_O_2_ which was determined as µmol g^−1^ fresh weight [66]. Production of O_2_^●−^ was estimated using the sulfanilamide method by determining the reaction at 530 nm. O_2_^●−^ production rate was recorded from a standard curve of NaNO_2_ reagent [67].

### 2.13. Statistical Analysis

Analysis of variance (ANOVA) procedures was done [68] using the MSTAT-C Statistical Software package. The means were compared by Duncan (1955) [69] when the difference was significant (*p* ≤ 0.05).

## 3. Results

### 3.1. Effects of Si and Proline on Morphological Characters in Sugar Beet Plants under Drought

**Figure 1:**
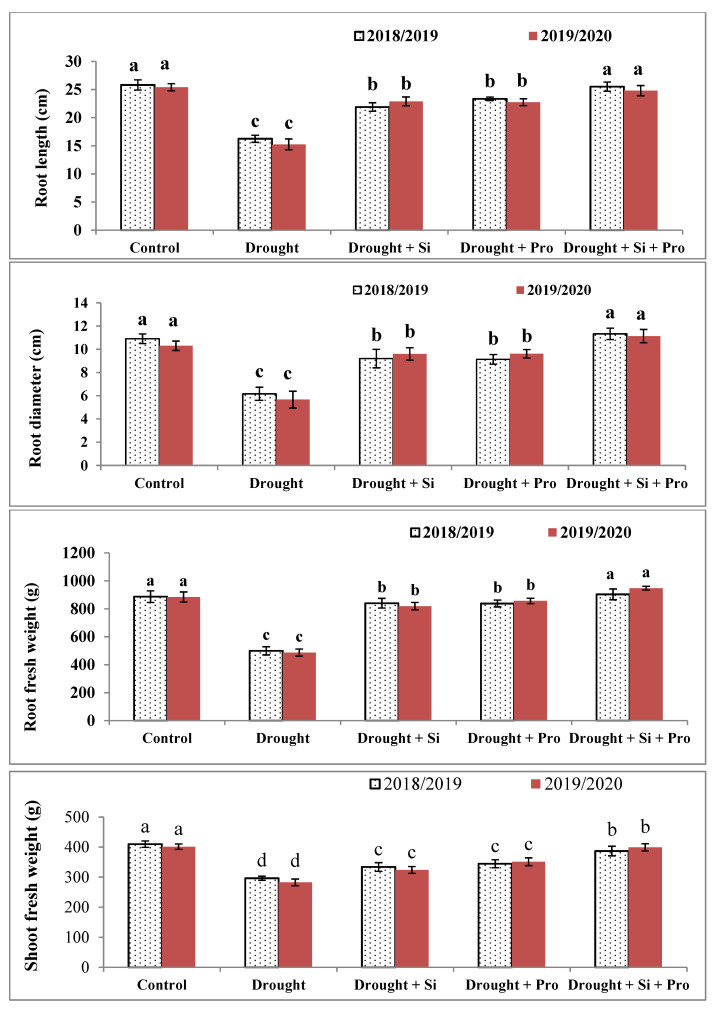
Effect of silicon and proline on root length (**A**), root diameter (**B**), root fresh weight (**C**) and shoot fresh weight (**D**) of sugar beet plants under drought in the 2018/2019 and 2019/2020 seasons. Bars followed by different letters are significantly different according to Duncan’s multiple range tests (DMRTs) at *p* < 0.05. Si: Silicon, Pro: Proline.

We observed that root length and diameter (cm), root and shoot fresh weights (g plant^−1^) of sugar beet plants under drought were considerably decreased (37, 40.9, 44.4 and 33.3%) compared with control plants (Figure 1A–D) as the main of the two seasons. However, application of Si or proline individually or combined causes a remarkable increase in root length and diameter, root and shoot fresh weights compared with drought-stressed untreated sugar beet plants in both seasons. Interestingly enough, that combination of Si + proline gives the maximum results of the above-mentioned traits (root length and diameter and root fresh weight in Figure 1A–C) without a difference when compared with control during both seasons.

### 3.2. Effects of Si and Proline on TSS%, Sucrose%, Root and Sugar Yield (t ha^−1^) in Sugar Beet Plants under Drought

**Figure 2:**
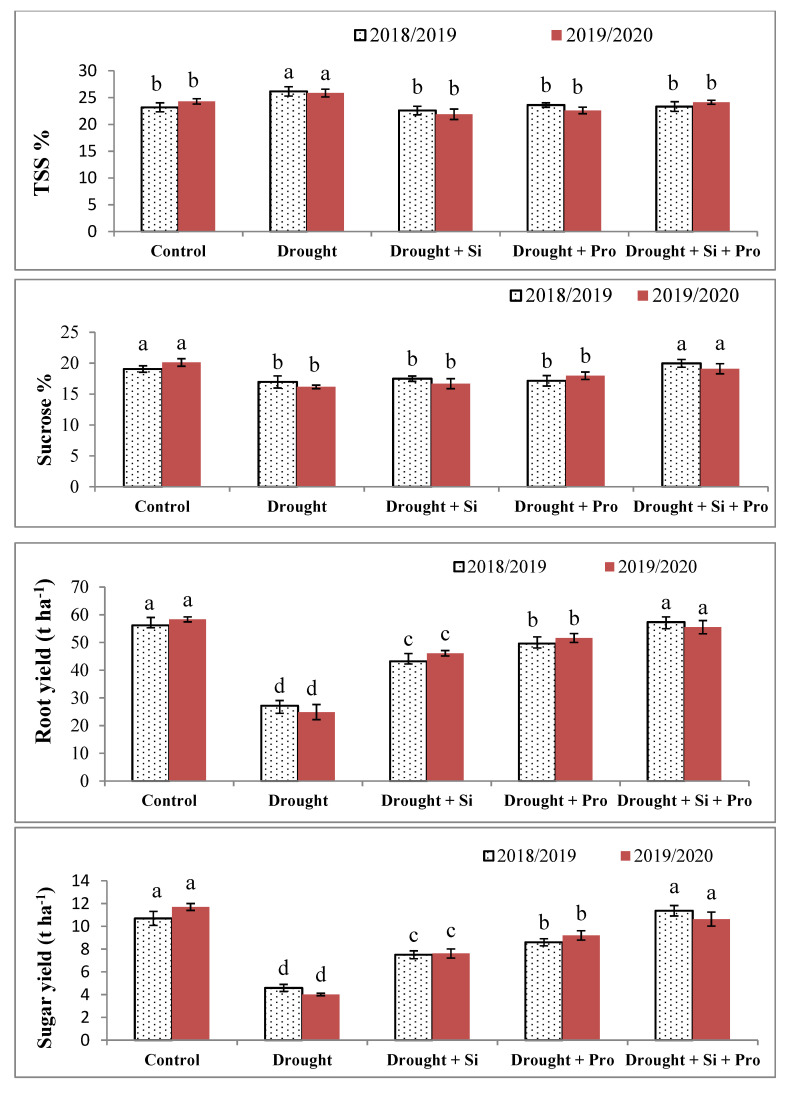
Effect of silicon and proline on TSS% (**A**), Sucrose% (**B**), root yield (**C**) and sugar yield (**D**) of sugar beet plants under drought in the 2018/2019 and 2019/2020 seasons. Bars followed by different letters are significantly different according to Duncan’s multiple range tests (DMRTs) at *p* < 0.05. Si: Silicon, Pro: Proline.

It can be noticed from Figure 2A–D that Total soluble solids, sucrose%, root yield and sugar yield of sugar beet plants were considerably affected under drought. TSS% significantly increased in sugar beet plants under drought (21.7%) compared with control and the other treatments in drought-stressed treated plants (Figure 2A) in both seasons. Furthermore, the application of Si + proline causes a significant reduction in TSS% compared with drought-stressed untreated plants (11%), however, the differences were not significant when compared with control treatment in the two seasons. Contrariwise, sucrose% was considerably decreased under drought in sugar beet plants in both seasons compared with control (19%). Additionally, Si or proline individually showed no significant difference in sucrose% in stressed plants compared with untreated stressed plants. Si + proline treatment gave the maximum results in sucrose% without differences when compared with control treatment (Figure 2B). The data presented in Figure 2C,D indicated that root and sugar yield was dramatically decreased in sugar beet plants under drought during two seasons. Nevertheless, sucrose% was considerably elevated as a result of Si or proline application individually or in combined and the best treatment was Si + proline without any significant difference with control treatment followed by proline then Si treatment during two seasons.

### 3.3. Effects of Si and Proline on Chl a (A), Chl b (B), RWC% (C) and EL% (D) in Sugar Beet Plants under Drought

**Figure 3:**
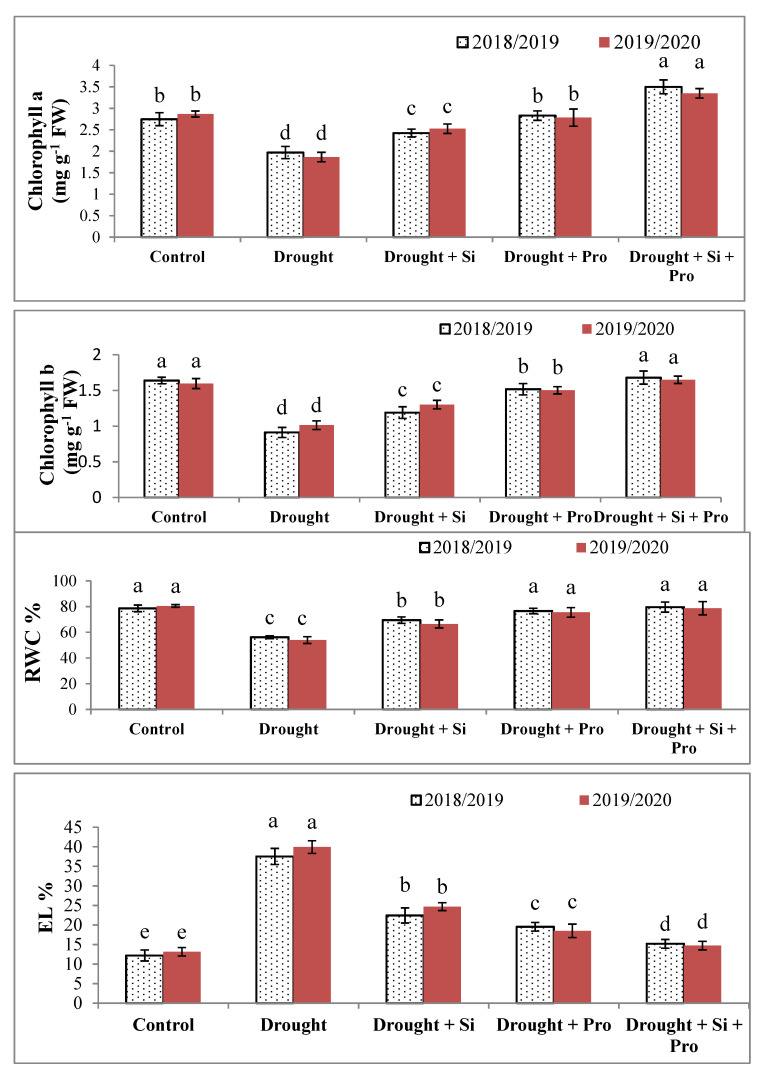
Effect of silicon and proline on Chlorophyll a (**A**), Chlorophyll b (**B**), relative water content (RWC)% (**C**) and electrolyte leakage (EL)% (**D**) of sugar beet plants under drought in 2018/2019 and 2019/2020 seasons. Bars followed by different letters are significantly different according to Duncan’s multiple range tests (DMRTs) at *p* < 0.05. Si: Silicon, Pro: Proline.

The obtained data in Figure 3A–D showed a remarkable reduces in Chl *a*, *b* concentrations and RWC in sugar beet plants under drought (22.2, 41.7 and 27.5%) compared with control. relative water content considerably decreased in sugar beet plants under drought compared to control, while electrolyte leakage% significantly elevated in stressed sugar beet plants in both seasons as compared to control (171.4%). In the present study, application of Si or proline or Si + proline significantly increased Chl a and b as compared to untreated stressed plants during two seasons. When compared with control there was no significant difference in Chl a with proline treatment, however, the maximum value of Chl a was achieved with Si + proline treatment in comparison to control and other treatments (Figure 3A). Si + proline treatment gave the maximum value of Chl b without any significant difference compared to control (Figure 3B).

The results of the current study in Figure 3C showed that RWC considerably elevated under drought in all treatments compared with untreated stressed sugar beet plants. The best results were recorded with proline then, Si + proline without any significant difference when compared with control treatment during both seasons (Figure 3C). Regarding EL%, data presented in Figure 3D indicated that El% dramatically decreased due to the application of Si or proline or Si + proline and the best treatment was Si + proline which, causes the best result and most decrease in EL% in comparison to other both treatment and stressed untreated sugar beet plants during both seasons.

### 3.4. Effects of Si and Proline on Proline Content (A), MDA (B), CAT Activity (C) and SOD Activity (D) in Sugar Beet Plants under Drought

**Figure 4:**
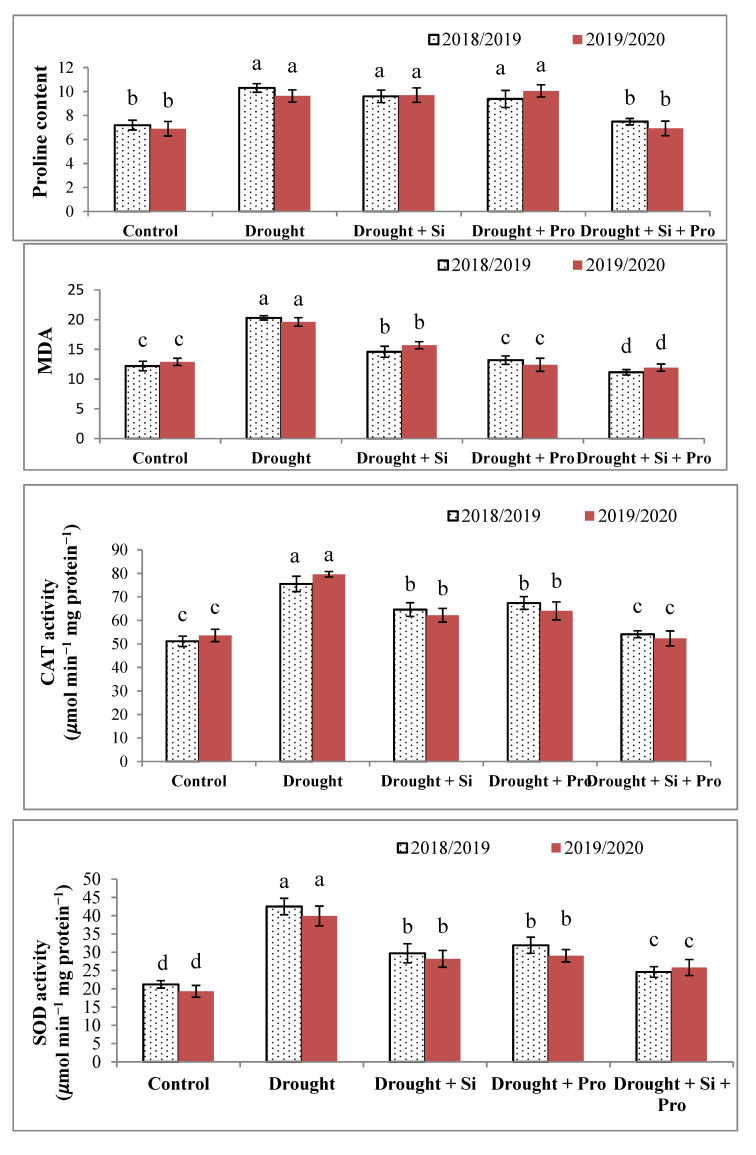
Effect of silicon and proline on proline content (**A**), lipid peroxidation (MDA) (**B**), antioxidant enzymes catalase (CAT) activity (**C**) and superoxide dismutase (SOD) activity (**D**) of sugar beet plants under drought in the 2018/2019 and 2019/2020 seasons.

Our results in Figure 4A demonstrated that proline content considerably elevated in plants under drought (38.5%) compared with control in the two seasons. Application of Si or proline led to increasing proline content in stressed plants without significant difference when compared with stressed untreated sugar plants, however, Si + proline treatment causes a remarkable reduction in proline content when compared with untreated stressed plants. However, there was no significant difference between Si + proline treatment and control. In addition, drought stress elicited a significant increase in MDA in drought-stressed sugar beet compared with control plants (60%). The helpful impact of Si or proline or Si + proline on decreasing oxidative stress and MDA was observed in Figure 4B, these treatments led to a remarkable decrease in MDA and the best treatment was Si + proline followed by proline then Si treatment during both seasons.

Antioxidant enzymes CAT and SOD activity as an indicator of stress, was more evident in sugar beet plants under drought compared with control. The presented data in Figure 4C,D showed that the antioxidant enzyme CAT and SOD activity considerably elevated (47.1 and 105%) in sugar beet plants under drought during both seasons. Si or proline or Si + proline effectively up-regulated CAT and SOD activities in sugar beet plants under drought. The best results of CAT and SOD activity were recorded with Si + proline treatment compared with untreated stressed plants during both seasons.

### 3.5. Effects of Si and Proline on Nitrogen(A), Phosphorus(B), Potassium (C) and Total Phenolic Compounds (D) in Sugar Beet Plants under Drought

**Figure 5:**
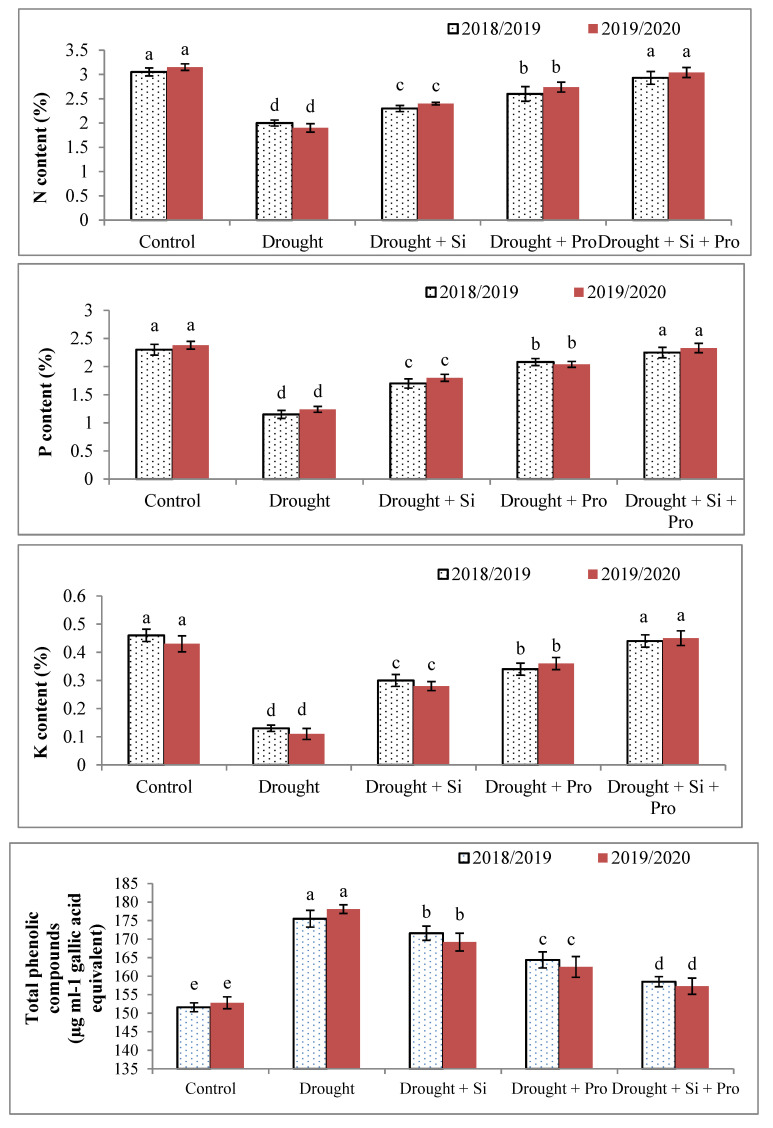
Effect of silicon and proline on nitrogen (**A**), phosphorus (**B**), potassium (**C**) and total phenolic compounds (**D**) of sugar beet plants under drought in the 2018/2019 and 2019/2020 seasons.

Drought stress considerably reduced Nitrogen, Phosphorus and Potassium (NPK) contents in sugar beet plants under drought during both seasons (Figure 5A–C). However, the results showed that application of Si or proline or Si + proline significantly increased NPK contents in sugar beet plants under drought compared with untreated stressed plants (38.7, 47.8, and 71.1%). Application of Si + proline led to improve sugar beet plants exposed to drought and give the best results of NPK contents in stressed plants compared with other treatment especially control plants without significant deference in both seasons. The best treatment was Si + proline followed by proline. Regarding total phenolic compounds, the results in Figure 5D showed a remarkable increase in sugar beet drought-stressed plants (17.1%) in comparison to control plants. Conversely, total phenolic compounds were considerably reduced in sugar beet plants under drought treated with Si or proline or Si + proline in comparison to untreated stressed plants during both seasons, the control gives the best results followed by combined application of Si + proline. 

### 3.6. Effects of Si and Proline on O_2_^●−^ (A)and H_2_O_2_ (B) in Sugar Beet Plants under Drought

**Figure 6:**
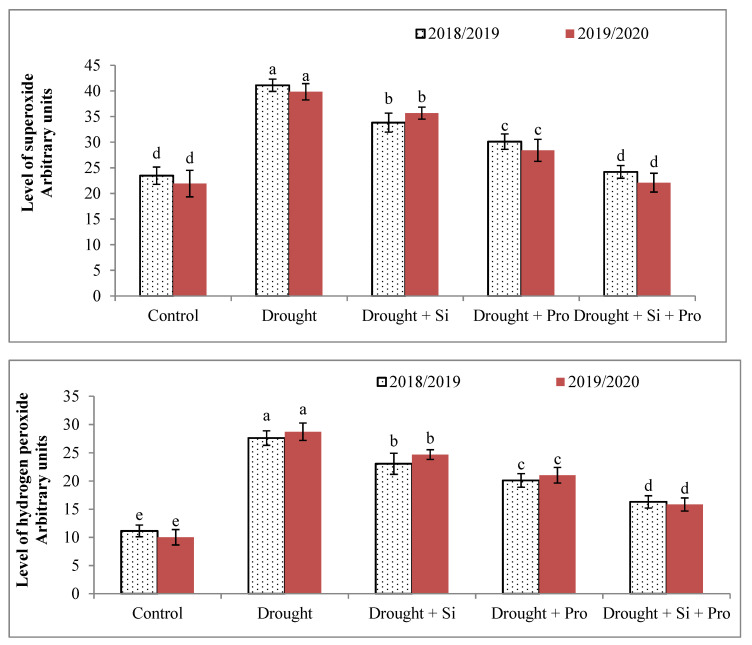
Effect of silicon and proline on Superoxide (O_2_^●−^) (**A**) and Hydrogen peroxide (H_2_O_2_) (**B**) of sugar beet plants under drought in the 2018/2019 and 2019/2020 seasons.

As a common response to drought, (O_2_^●−^) and (H_2_O_2_) dramatically elevated in sugar beet plants under drought during two seasons compared with control (Figure 6A,B). The achieved results presented that Si or proline or combined application of Si + proline led to a remarkable decrease in O_2_^●−^ and H_2_O_2_ levels in sugar beet plants under drought (86.9 and 163.6%) compared with untreated stressed plants. According to O_2_^●−^ level (Figure 6A), the lowest level as the best result was achieved with combined application of Si + proline in comparison to other treatments and without any significant difference with control. The application of Si + proline gave the lowest level of H_2_O_2_ compared with the application of Si or proline individually during both seasons (Figure 6B).

## 4. Discussion

### 4.1. Effects of Si and Proline on Morphological Characters

It is well known that drought detrimentally affects the growth and yield characters in the plants [13,16,18,39]. Our results displayed that drought causes a major decrease in root length and diameter (cm), root and shoot fresh weights (g plant^−1^) in sugar beet plants under drought during both seasons, this decrease because of the harmful effect of drought on water absorption during root system from the soil, consequently, reduced cell division, cell enlargement and decrease RWC. Additionally, drought negatively affects cell membrane and reduces growth parameters for example root diameter and length, root and shoots fresh weights. EL-Darder et al. [70] found that reducing the amount of irrigation water significantly declined the mean root of sugar beet. However, foliar application of Si or proline or the combination of Si + proline led to alleviate the negative impact of drought resulting in enhancement of sugar beet plant status and increase root diameter and length, root and shoot fresh weights. This increase because of the positive role of Si as a useful element in increasing the growth and development of plants under different stresses [8,49]. Similarly, Si is effective in alleviating drought stress by increasing water holding capacity, enhancing soil fertility and regulates stomatal conductance as well as the photosynthesis process [71]. In the current research, proline has a beneficial role in decreasing the damaging impact of drought on sugar beet, this useful impact of proline might be due to its role in protecting enzymes, proteins structures and membranes that helping plants to tolerate stresses [72]. These results were comparable to the results of El-Shawa et al. [9], Abdelaal et al. [38], Teh et al. [41].

### 4.2. Effects of Si and Proline on TSS%, Sucrose%, Root and Sugar Yield

The adverse impacts of drought on sucrose%, root and sugar yield were observed in drought-stressed plants during both seasons, this result of drought may be due to its negative impact on root diameter, root length and root fresh weight because of the decrease in water absorption, cell division, cell elongation and CO_2_ assimilation, consequently, decrease sucrose%. These results are in agreement with the obtained results with Foroozesh et al. [73] and Chołuj et al. [74], they reported that drought led to inhibit the assimilation of CO_2_ and reduce the assimilate supply in sugar beet, consequently, decrease sucrose%, root and sugar yield. Contrariwise, drought stress causes an increase in TSS% in sugar beet plants during two seasons. Furthermore, Si or proline or the combined application of Si + proline led to improve root and sugar yield and decrease TSS% especially, the combined application of Si + proline. The superior effect of Si + proline might be due to the role of proline in improving sugar beet as a storage sink for nutrient elements for example carbon and nitrogen and as a scavenger for free-radical consequently, decreased TSS% [75]. Si plays an important role in improving respiratory enzyme activity and decrease oxidative stress signals which are considerably accumulated under stress such as ROS, MDA and TSS% in sugar beet.

### 4.3. Effects of Si and Proline on Chlorophyll a, Chlorophyll b, RWC% (C) and EL%

Chlorophyll *a, b* concentrations and RWC considerably reduced due to drought in sugar beet compared with control during two seasons. According to Chl concentration, Hsu and Kao [76] found that Chl was decreased under drought, this adverse effect on Chl because of the osmotic stress, decreasing water holding capacity and stomatal movement which limits CO_2_ influx to leaves, decreasing photosynthesis, consequently, reduce Chl a and b concentrations. Additionally, the decrease of Chl concentration under drought might be due to the accumulation of ROS, resulting in Chl degradation by chlorophyllase enzyme, which increases the Chl degradation and the destruction of chloroplasts, also, drought led to a reduction in photosystem II activity and the rate of CO_2_ assimilation in sugar beet [74]. These results were consistent with the recorded results in sugar beet plants [19] and in barley plants under drought stress [38]. Contrariwise, EL% considerably increased in sugar beet plants under drought compared with control, this increase is due to the adverse impact of drought on sugar beet resulting in damage to the plasma membrane, dehydration of cytoplasm and membrane stability, this result was in line with those recorded in drought-stressed barley plants [38]. On the other hand, Chl *a, b* concentrations and RWC were increased significantly due to Si or proline or the combined application of Si + proline in sugar beet plants under drought in comparison to stressed untreated plants. This valuable effect of Si + proline could be due to that proline protect plasma membrane, cytoplasmic enzymes, stabilize membranes and proteins and inhibit ROS [28]. Si has a protective role against stress, it helps in increasing the concentration of Ca which plays an essential role in improving membrane stability and stimulates some enzymes to decrease ROS accumulation and improve electron transport chain [47,48]. With our findings, it was suggested that Si led to improve the carotenoids and chlorophyll content, produce antioxidant compounds, improve the gas-exchange process and Hill reaction [77,78].

### 4.4. Effects of Si and Proline on Proline Content, MDA, CAT Activity and SOD Activity

It is well-known that proline accumulation, over-expression of MDA content and CAT and SOD activities in sugar beet under drought displays a defense mechanism against the negative impacts of drought, the abovementioned characters considerably increased in stressed sugar beet plants in comparison to control. The over-accumulation in MDA and proline is a response to drought, our findings are in agreement with the results of some researchers, they reported that proline and MDA considerably elevated under stress circumstances in many plants [9,10,28,42]. CAT and SOD activities significantly elevated in sugar beet under drought compared with control, this increase may be due that CAT and SOD are antioxidant enzymes, which involved in the tolerance of various stresses, SOD is the first defense wall in oxidative damage in the cells and play a key role in alteration of O_2_^●−^ radicals to H_2_O_2_ and oxygen (O_2_) [79]. CAT participates in the conversion of H_2_O_2_ into H_2_O and oxygen, play a pivotal role in plant metabolism and in signal recognition These results are in line with the results of Abdelaal et al. [16,18,19,38] and Li et al. [80]. Our findings showed the valuable effects of Si or proline or the combined application of Si + proline on drought-stressed sugar beet compared with untreated stressed plants. The important impact of Si may be due to its role in improving electron transport chain and enzyme stimulation, this positive role of Si was reported in many plants [8,39,42].

Additionally, the pivotal role of prolin may be due to its useful effect as osmoprotectant in protecting the plant cells from oxidative stress by osmotic adjustment, protein stabilization and antioxidant enzyme balance [81]. Our findings are in line with the results of Abdelaal et al. [38], Ribera-Fonseca et al. [77] and Pontigo et al. [78].

### 4.5. Effects of Si and Proline on Nitrogen, Phosphorus, Potassium and Total Phenolic Compounds

Drought stress adversely affects nutrients content, mainly, NPK in stressed plants during both seasons compared with control. The adverse influence of drought on NPK could be due to the reduction in nutrient flow and transport under drought [81]. NPK are very important nutrients to plant, these nutrients are involved in many biochemical and physiological processes in the cells such as photosynthesis and stomatal movement. Contrariwise, total phenolic compounds significantly elevated in stressed plants under drought, these compounds naturally exist in plants and produced in the endoplasmic reticulum and cytoplasm, play an important role as signal molecules, scavenge ROS and act as secondary antioxidant protection system under stress conditions [82]. Regarding to the impact of Si or proline or the combined application of Si + proline on drought-stressed sugar beet plants, the results exhibited helpful effects of these treatments and led to a significant increase in NPK contents in stressed plants. The application of these treatments led to regulate total phenolic compounds formation in sugar beet under drought in comparison to untreated stressed plants. The remarkable increases in NPK was recorded in the current research due to Si+ proline application, this increase might be due to the role of Si in improving membrane H ± ATPase activity which enhances element uptake, mainly K^+^ and Ca^+^ and improve photosynthesis and water relations [83]. The helpful impact of Si on nutrient content was observed in some plants [8,42] and that could be explained by the regulation of key enzyme activity in the phenylpropanoid pathway [84] and the improvement of total phenol formation [85]. The role of proline in increasing NPK content under drought could be due to that proline is an amino acid and involved in increase plant tolerance to stresses by enhancement plant metabolism as well as increase nutrients uptake [86] as well as increase energy production in the electron transport chain and ATP synthesis [87].

### 4.6. Effects of Si and Proline on Superoxide (O_2_^●−^) and Hydrogen Peroxide (H_2_O_2_)

ROS are produced under stressful and normal conditions at different cellular sites, principally, peroxisomes, mitochondria and chloroplasts, ROS accumulation depends on the release of electrons onto O_2_ coming from the electron transport chain in mitochondria, chloroplasts and plasma membranes. ROS over accumulation especially O_2_^●−^ and H_2_O_2_ were observed in sugar beet under drought in comparison to control. This increase in O_2_^●−^ and H_2_O_2_ may be a result of the adverse effect of drought on sugar beet resulting in oxidative damage to mitochondria, chloroplasts, membrane and cytotoxicity in plants. The over-accumulation of O_2_^●−^ and H_2_O_2_ is one of the central responses under various stresses [88,89], this accumulation may cause disturbance in the nucleic acid conformation, lipid peroxidation and proteins oxidation, finally, the programmed cell death [90]. In the current research, we recorded a considerable decrease in O_2_^●−^ and H_2_O_2_ because of foliar treatment with Si or proline or the combined application of Si + proline. Si + proline application was more active in alleviating the harmful impact of drought and decreasing O_2_^●−^ and H_2_O_2_ levels in sugar beet under drought compared with stressed untreated plants. This significant effect of Si + proline may be due to the role of Si in antioxidant enzyme stimulation, improvement of Chl concentration and decrease O_2_^●−^ and H_2_O_2_ formation [38,48]. Si is involved in the decrease of ROS formation under abiotic stresses, this reduction of ROS levels leads to improve photosynthesis and enhance the plant immune system under negative conditions. The increase of antioxidative compounds might decrease the adverse effects of ROS and increase plant tolerance [8,38,91]. Furthermore, proline is one of the major osmolytes, many plants synthesize proline to improves membrane stability, tolerate osmotic stresses by decreasing ROS formation and ROS scavenging [92]. Under drought stress, proline protects cell membranes, cytoplasmic enzymes, proteins and scavenges ROS. In general, our study revealed that the negative impact of drought on sugar beet plants could be alleviated by Si or proline or the combined application of Si + proline, these treatments cause a decrease in oxidative damage, regulate proline and total phenol compounds as well as enhance the activity of antioxidant enzymes, increase Chl concentration, consequently, improve yield parameters.

## 5. Conclusions

Drought stress considerably reduced growth, root yield, sugar yield and sucrose% of sugar beet mainly due to oxidative stress. Nevertheless, antioxidant enzyme activities were elevated under drought to induce plant defense system and scavenge O_2_^●−^ and H_2_O_2_. Under drought, Chl a, Chl b, RWC and NPK content significantly reduced but, total phenolic compounds, EL and ROS were considerably elevated in sugar beet plants. However, the combined application of Si + proline led to a decrease in the detrimental impacts of drought and improve Chl concentration, RWC, NPK contents, regulate the activity of CAT and SOD enzymes and increase yield parameters of sugar beet plants. Current study findings concluded that application combined of Si + proline (2 mmol Si + 10 mmol proline) has confirmed to be effective in mitigating drought stress damages in sugar beet plants.

## Data Availability

Not applicable.
